# Safety sign comprehension of fiberboard industry employees

**DOI:** 10.1016/j.heliyon.2023.e16744

**Published:** 2023-05-26

**Authors:** Celal Gungor

**Affiliations:** Department of Forest Industrial Enginnering, Faculty of Forestry, Izmir Katip Celebi University, Izmir, Turkey

**Keywords:** Safety, Safety signs, Fiberboard industry, Sign comprehension, Accident prevention

## Abstract

Safety signs are very important communication tools for accident prevention, fire safety, health hazard information, and emergency evacuation. They are helpful when properly designed and understood by employees. The purpose of the present study was to investigate fiberboard industry employees' understanding of safety signs. 139 participants were asked to indicate the meaning of a series of 22 commonly used safety signs. The mean comprehension score for 22 signs was 66.6% (min. 22.5% and max. 98.6%). The mean score for warning signs was the lowest, prohibition signs was the highest. Poor comprehension score (less than 40%) was noted for the toxic material, automated external heart defibrillator, overhead obstacle, and disconnect mains plug from electrical outlet signs. These low comprehension scores indicate that some symbols may not effectively convey the message to the audience. Safety practitioners and trainers should pay more attention to teach the actual meaning of those signs.

## 1 Introduction

Safety signs are important because they help employees identify potential hazards and inform them how to avoid those hazards. When signs convey the message correctly, they can prevent occupational accidents and illnesses. The effect of characteristics of the person interacting with the sign on comprehension performance has been studied by several researchers. For example, studies [[1], [2], [3], [4], [5], [6]] found that older participants performed significantly worse than younger participants. Educational level [7,8] and prior training [3,[9], [10], [11], [12], [13]] also improved comprehension levels. The effects of cultural differences on comprehension of graphic symbols have been demonstrated by several researchers [[14], [15], [16], [17], [18], [19], [20], [21], [22], [23], [24]]. The design characteristics of a sign (i.e., color, shape, pictogram, size, visibility, readability, and comprehensibility, as well as the placement, illumination, and maintenance of the sign) and working conditions (i.e., dust, chemicals and fumes in the air, precipitation, time pressure, and performing multiple tasks simultaneously) can also affect the comprehensibility of a sign.

Workplaces in the fiberboard industry (16.21 NACE Code, the European statistical classification of economic activities Code “Nomenclature of Economic Activities” for Manufacture of veneer sheets and wood-based panels [25]) are classified as "very hazardous" workplaces by Turkish legislation [26] because there are significant physical, chemical, biological, ergonomic, and psychosocial hazards in the industry. For example, physical (noise, vibration, and heat stress) and mechanical (slip, trip and falls, moving machinery, electrical equipment) hazards, chemical hazards due to physical, chemical and toxic properties of the chemicals and dusts, psychosocial hazards (fatigue, stress, work pressure, and lack of training), and ergonomic hazards due to repetitive movements, awkward posture, and prolonged duration of work are some common hazards in the fiberboard manufacturing industry. Proactive safety activities, including engineering and management control measures such as posting appropriate signs and conducting training on the signs, are critical to prevent or minimize occupational injuries, illnesses, and fatalities resulting from these hazards. It should be noted that workplaces belonging to the "manufacture of veneer sheets and wood-based panels" (16.21 NACE Code), including the manufacture of medium-density fiberboard (MDF) and other fiberboard industries, are classified as "very hazardous" in the Legal Notice on Workplace Occupational Health and Safety Related Hazard Classes. If a workplace is in the "very hazardous" hazard class, each employee in that workplace must receive at least 16 h of occupational health and safety training each year. Most training includes a 1-h course on safety signs.

All United Nations (UN) Member States adopted seventeen Sustainable Development Goals in 2015 [27]. The 8th of these goals is about promoting sustainable economic growth, employment and decent work for everyone, everywhere. The specific goal is to protect labor rights and promote safe and secure working environments for all workers by 2030. The present study aims to investigate the level of subjective comprehension of safety signs among fiberboard industry workers in Turkey. The study seeks to identify poorly comprehended signs and also to understand what affects safety signs comprehension. A better understanding of sign design issues can enable safety professionals to create safer workplaces, as UN aims to do.

## 2 Materials and methods

### 2.1 Sample

Participants in the present study were recruited from a fiberboard factory in western Turkey. The research population includes all employee positions, including managers, engineers, technicians (foremen, mechanics, etc.), operators (production operators, vehicle operators, repair and maintenance operators, etc.), and other employees (service providers, etc.). All participants were Turkish and their native language was Turkish. There was a total of 341 employees in the plant. They were all sent two emails (one invitation and one reminder) to participate in an online survey. A total of 163 subjects responded to the survey. Six participants did not agree to the consent form and 18 subjects either did not provide demographic information or did not complete the survey. Thus, a total of 139 subjects voluntarily participated in the study, yielding a 40.8% participation rate.

All subjects were given a consent form informing them of the purpose of the study, the procedures, and the potential risks and benefits of their participation. Subjects were required to read and approve the consent form before answering any questions. It should be noted that all ethical principles, codes, and guidelines of the Belmont Report [28], the Nuremberg Code [29], and the Declaration of Helsinki [30] were carefully followed by the researcher to protect the free will, privacy, confidentiality, and well-being of the subjects. The study was approved by the Social Sciences Ethics Committee of Izmir Katip Celebi University (the number of the ethical approval: SAE -0215-2021/23-01).

### 2.2 Data collection

An online questionnaire was used to assess the understanding of safety signs by employees in the fiberboard industry. The questionnaire consists of two sections. The first section asked subjects about their demographics and background knowledge of occupational safety and health. The second section contained multiple-choice test questions on the meaning of safety signs. A set of twenty-two safety signs prescribed by the International Organization for Standardization (ISO 7010) was used [31]. Five of them were prohibition signs (P011, P014, P020, P041, and P048), five of them were mandatory action signs (M005, M006, M012, M021, and M029), five of them were warning signs (W003, W016, W018, W020, and W024), six of them were evacuation routes, locations of safety equipment or safety facilities, safety action signs (E007, E008, E010, E012, E015, and E034), and one was a fire equipment and fire action sign (F016). These signs were randomly selected among signs commonly used in the fiberboard industry. Each multiple-choice question had one correct and three incorrect (but plausible) answer choices as adopted by Refs. [32,33]. Subjects were asked to select only one choice that they believed to be true. They were asked to answer all questions even if they did not know the sign. They were explained that the research interest was their interpretation of the signs. A list of 22 signs were displayed to the participant of the study. The size of the signs displayed was 2.3 cm × 2.3 cm. The safety signs were displayed in color. As mentioned in the standard ISO 7010 [31], the shape and color of each safety sign conforms to the standard ISO 3864-1 [34] and the design of the graphic symbols conforms to the standard ISO 3864-3 [35].

### 2.3 Statistical analyses

Descriptive statistics were calculated on subjects' demographics and their self-reported knowledge of occupational safety and health. Independent samples t-tests for two groups (i.e., combined experience and education levels) and the Analysis of Variance (ANOVA) tests for more than two groups (i.e., age and position in the workplace) were performed to test whether there were significant differences between subjects' comprehension performance and characteristics. Pearson's chi-square statistical tests were applied to determine the relationship between subjects' demographic characteristics and correct response to a sign. The significance level for this study was set at 0.05. All statistical analyzes were performed using IBM SPSS Statistics for Windows, Version 22.0.

### 2.4 Comparison of scores

Results of the present study were compared with acceptance criteria in the standards. A 67% score of comprehension is used as the acceptance criterion for safety-related symbols in ISO 3864 [34,35] and an 85% score of comprehension is used as an acceptance criterion in the American National Institute (ANSI) Z535.3–2022 [36].

## 3 Results and discussion

### 3.1 Demographic data and background of study subjects

Subjects were asked to provide their demographic data and level of knowledge about occupational safety and health. The demographic data of the subjects are shown in Table 1. Almost all subjects (99.3%) were male. The average age of all subjects was 32.15 (SD = 4.8) years and 87% of subjects were between 26 and 40 years old. The majority of subjects were technical employees. The total work experience averaged 9.04 years with a standard deviation of 4.9 years. Most subjects (61.7%) had experience between 6 and 15 years.

**Table 1:** Demographic data of the study participants.

Variable	Category	n	%
Gender (N = 139)	Male	138	99.3
	Female	1	0.7
Age (years) (N = 139)	≤25	10	7.2
	26–30	39	28.0
	31–35	63	45.3
	36–40	19	13.7
	≥41	8	5.8
Educational level (N = 138)	Elementary school	2	1.4
	High school	53	38.4
	Associate’s degree	44	31.9
	Bachelor’s degree	35	25.4
	Master’s degree	4	2.9
Position (N = 138)	Manager	7	5.1
	Engineer (engineer, chief or senior engineer)	28	20.3
	Technician (foreman, mechanic, etc.)	16	11.6
	Operator (maintenance, production, etc.)	81	58.7
	Other employee (general service employees etc.)	6	4.3
Total work experience (years)	1–5	40	28.8
	6–10	61	43.9
	11–15	26	18.7
	≥16	12	8.6

Subjects were asked if they had ever received training on health and safety signs. 116 subjects (83.5%) reported that they had received training in the workplace. 12 subjects (8.6%) indicated that they had received a total of 220 h of additional training in order to sit for the Occupational Health and Safety Expert exam (the certificate is awarded by the Turkish Ministry of Labor and Social Security). 4 subjects (2.9%) received some type of training other than these two trainings. 7 subjects (5.0%) had not received any training at all.

Subjects were also asked to rate their level of knowledge about occupational health and safety on a 5-point Likert scale (1: very low level of knowledge, 2: low level of knowledge, 3: medium level of knowledge, 4: high level of knowledge, 5: very high level of knowledge). Of the 138 subjects who rated their knowledge level, 2 subjects (1.5%) rated their knowledge level as very low and 5 subjects (3.6%) rated their knowledge level as low. 44 subjects (31.9%) rated their knowledge level as medium, 69 subjects (50.0%) as high, and 18 subjects (13.0%) as very high.

### 3.2 Subjects’ comprehension levels on safety signs

Subjects were asked about the meaning of each safety sign. The distribution of the subjects' responses can be found in Table 2. Note that the order of signs and answers is given in the order in the questionnaire. The overall mean comprehension score for twenty-two safety signs was 66.6% with a standard deviation of 24.2%, indicating a moderate level of comprehension in accordance with the ISO standard. The comprehension scores for each sign ranged from the lowest score of 22.5% to the highest score of 98.6%. More than half of the safety signs (59.1%) achieved a comprehension score of 67% or higher which is established as the acceptance criterion for safety-related symbols in ISO 3864 [34,35]. Of twenty-two signs, five signs (22.7%) achieved a comprehension score greater than 85%, which is specified as an acceptance criterion in ANSI Z535.3–2022 [36]. For five signs, the mean comprehension score ranged from 40% to 67%, and for four signs it was even lower than 40%.

**Table 2:** Subjects’ responses on the meaning of the safety sign. Note: Correct answers are in bold and italics.

ISO 7010 Number	Sign	Choices for the meaning of the sign	n	%
W020		Do not replace the bulb unless you are authorized	8	5.8
		Excessive light source	28	20.4
		Falling object hazard	53	38.7
		***Overhead obstacle***	**48**	**35.0**
		Missing	2	
E034		Slide equipment to move	8	5.8
		Refrigerator can move	–	–
		***Door slides left to open***	**66**	**47.5**
		Exit door is on the left side	65	46.8
		Missing	–	
M021		***Disconnecting the machine or equipment before carrying out maintenance or repair***	**114**	**84.4**
		The train changes track	4	3.0
		Pull the handle to stop	6	4.4
		Change the line	11	8.1
		Missing	4	
P041		Go back to work	–	0.0
		No pushing	3	2.2
		Waiting is forbidden here	20	14.5
		***No leaning against***	**115**	**83.3**
		Missing	1	
W016		Electrical hazard	3	2.2
		Danger of death	101	73.2
		High voltage	3	2.2
		***Toxic material***	**31**	**22.5**
		Missing	1	
E007		***Evacuation assembly point***	**137**	**98.6**
		Attention, there are people nearby	2	1.4
		Entry is prohibited. Only certain people can enter.	–	0.0
		Enter with a group	–	0.0
		Missing	–	
M012		Stairs	7	5.1
		Escalator	51	37.0
		***Use handrail***	**66**	**47.8**
		Inclined road	14	10.1
		Missing	1	
F016		Prevent the spread of fire	29	21.2
		Protect yourself from fire	32	23.4
		***Location of a fire blanket***	**73**	**53.3**
		It is forbidden to approach the fire area	3	2.2
		Missing	2	
E012		Take a shower before entering the building	16	11.5
		***Location of a safety shower***	**123**	**88.5**
		Do not use this device in a bathtub, shower, or water-filled reservoir	–	0.0
		Caution, you may get wet	–	0.0
		Missing	–	
W018		Low temperature/freezing conditions	1	0.7
		***Machinery may start automatically***	**56**	**40.9**
		Heavy wind	32	23.4
		Biological hazard	48	35.0
		Missing	2	
P011		No open fire	10	7.2
		No fire	6	4.3
		***Do not extinguish with water***	**116**	**84.1**
		Do not extinguish the fire	6	4.3
		Missing	1	
M006		It is forbidden to insert the plug into the outlet	3	2.2
		***Disconnect mains plug from the electrical outlet***	**54**	**38.8**
		When pulling the plug from the outlet, pull the plug, not the cord	54	38.8
		Disconnect the plug before carrying out maintenance or repair	28	20.1
		Missing	–	
P014		Choking hazard	12	8.8
		No reaching in	7	5.1
		***No access for people with metallic implants***	**112**	**82.4**
		Crushing of hands	5	3.7
		Missing	3	
E015		Turn off the tap after use	22	16.1
		Hot water	6	4.4
		***Location of drinking water***	**99**	**72.3**
		Not drinking water	10	7.3
		Missing	2	
P020		***Do not use lift in the event of fire***	**132**	**95.0**
		Do not use the lift for more than one person	4	2.9
		Do not use the lift for people	–	0.0
		Do not approach with open flame	3	2.2
		Missing	–	
W003		Aircraft flight area	4	2.9
		Chemical spill hazard	2	1.4
		***Radioactive material or ionizing radiation***	**131**	**94.2**
		Biological hazard	2	1.4
		Missing	–	
M029		***Sound horn***	**109**	**78.4**
		Do not sound horn	3	2.2
		Caution, very high horn sound	25	18.0
		Horn can be played at certain times	2	1.4
		Missing	–	
E008		Clear the glass	7	5.1
		Glass breakage hazard	42	30.7
		Holding the bar is prohibited	21	15.3
		***Break to obtain access***	**67**	**48.9**
		Missing	2	
P048		***Do not run***	**124**	**89.2**
		No pedestrian	10	7.2
		No thoroughfare/trespassing	5	3.6
		Use this walkway	–	0.0
		Missing		
W024		No reaching in	28	20.1
		Put your hand inside	9	6.5
		Do not touch	–	0.0
		***Crushing of hands***	**102**	**73.4**
		Missing	–	
M005		***Connect an earth terminal to the ground***	**111**	**79.9**
		Heavy load (dumbbells)	9	6.5
		High radiation area	2	1.4
		Strong signal area	17	12.2
		Missing	–	
E010		***Location of an automated external heart defibrillator device***	**37**	**27.4**
		High voltage power can cause your heart to stop	55	40.7
		No access for people with active implanted cardiac devices	20	14.8
		Check the power level of your implanted cardiac device	23	17.0
		Missing	4	

The overall mean (66.6%) of comprehension score in the present study was similar to some previous studies (63.4% [8], 63.8% [19], 66.2% [37], 67.5% [7], 69.2% [38], and 70.9% [39]). On the other hand, Davoudian Talab and Azari [40] reported a higher overall rate of perception (78.4%) and Arphorn et al. [14] very low scores in safety sign perception (39.2%). The differences may be due to different backgrounds (e.g., ethnic, cultural, educational) or different experimental designs (e.g., sign selection). Some previous studies [17,[19], [20], [21], [22], [23]] indicated that different cultural backgrounds might lead to differences in sign perception.

The mean comprehension score for five warning signs (W003, W016, W018, W020, and W024) in the present study was 53.2%. Three warning signs were among the five lowest comprehension scores. The literature [37,41] reported a mean score of 61.2% and 63.4% for the warning signs, respectively, which is slightly higher than the present study. The lowest comprehension score (22.5%) in the present study was for the sign "Toxic material" (W016). The majority of subjects (73.2%) thought it was a sign that read "Danger of death." This may be due to the cultural background of Turkish employees. A skull and two crossed bones on the back might be thought as death. Moreover, in the past decades, almost all covers of electrical control panel in Turkey carried this sign with the explanatory text "Danger of death." The wrong use of the sign in the past could prevent them from learning the new/real meaning. For the same sign, Akpinar et al. [42] (another Turkish study), Ng et al. [7] and Davoudian Talab and Azari [40] reported lower or similar compression scores as in the present study. But Zamanian et al. [39] reported a 92.5% comprehension score for the same sign. Another warning sign with a very low comprehension score (35.0%) was the sign “Overhead obstacle” (W020) sign. Most subjects responded with “Falling object hazard” (38.7%) or “Excessive light source” (20.4%). Ng et al. [7] used a different sign for the overhead obstacle hazard sign in their study and also received a lower score (less than 50%). The sign “Machinery may start automatically” (W018) sign also had a low comprehension score (40.9%). The message of the sign is to warn employees about automatic activation of machinery or moving mechanical parts. However, 35.5% of subjects thought it was a sign for biological hazards and 23.4% of subjects thought it was a sign for heavy wind. On the other hand, the sign “Radioactive material or ionizing radiation” (W003) was answered correctly by 94.2% of the subjects. Some previous studies [7,37,43] also reported high comprehension scores for the radiation hazard sign.

There was only one safety sign for fire safety. The sign for "Location of a fire blanket" (F016) was answered correctly by 53.3% of the subjects. 23.4% of the subjects thought that it was used to protect themselves from fire and 21.2% thought that it was used to prevent the spread of fire. Based on the human figure holding a fire blanket and the flame figure on the sign, subjects might think that it is a mandatory action even though it is not blue in color and circle in shape.

The mean comprehension score was 63.9% for six evacuation route, location of safety equipment or safety facility, safety action signs (E007, E008, E010, E012, E015, and E034). The sign "Evacuation assembly point" (E007) had the highest comprehension score (98.6%) in the present study. Only two subjects thought it was "Attention, there are people nearby." Since Turkey is located in several active earthquake zones, people in Turkey are well trained for emergencies and there are many places (not only workplaces but also all public places) that are designated as evacuation assembly points. These places are marked with "Evacuation assembly point" signs. This may be the reason why almost all subjects know its meaning of these signs. In contrast, the sign "Location of an automated external heart defibrillator device" (E010) had one of the lowest comprehension scores in the study. Only 27.4% of subjects knew its meaning. This means that 72.6% of subjects could not identify the location of the external heart defibrillator device when it was needed. Many subjects (40.7%) thought that this sign indicated the high voltage hazard and that high voltage could cause cardiac arrest. Another sign, "Door slides left to open" (E034), was also misunderstood by 52.6% of subjects. It can be assumed that 46.8% of subjects would not slide the door to evacuate the building, but would continue to the left to find an exit door. Another emergency sign with a low comprehension score (48.9%) was "Break to obtain access" (E008). 30.7% of the subjects thought it was a hazard sign for broken glass, although the hazard sign should be yellow in color and triangle in shape.

The mean score of the five mandatory action signs (M005, M006, M012, M021, and M029) was 65.9%, which is close to the acceptance criterion (67%) for safety-related symbols of ISO 3864, but lower than in the study of Tam et al. [41] (87.9%). The sign "Disconnect mains plug from the electrical outlet" (M006) was answered correctly by 38.8% of the subjects. Another 38.8% of subjects thought it meant "When pulling the plug from the outlet, pull the plug, not the cord." 20.1% of subjects confused this sign with "Disconnect the plug before carrying out maintenance or repair" (M021). The comprehension score of this sign (M021) alone was 84.4%. The sign "Use handrail" (M012) was misunderstood as an escalator by 37.0% of the subjects and as an inclined road by 10.1%.

The highest mean score in the present study was obtained for the group of prohibition signs (86.8%), which is higher than the acceptance criterion of ISO and some studies in the literature [37,41]. Comprehension scores for "No access for people with metallic implants" (P014), "No leaning against" (P041), "Do not extinguish with water" (P011), "Do not run" (P048), and "Do not use lift in the event of fire" (P020) were 82.4%, 83.3%, 84.1%, 89.2%, and 95.0%, respectively.

It should be beneficial to mention in here that even if the answer is formally incorrect, the signal may convey to the employees a meaning of hazard. It can also mean that employees do not completely misunderstand the sign. However, it should be noted that knowing a sign indicating a hazard is not enough and that it is necessary to know the specific hazards and how to respond to them.

It is also worth noting that comprehension errors of safety signs would be more critical for some signs than others, depending on the level of risk involved. The level of risk is determined by the likelihood and severity of harm or adverse health effects that may result from exposure to a hazard. Therefore, more attention should be paid to signs that convey higher risks to ensure effective communication and minimize the likelihood of injuries and illnesses.

Some previous studies [41,44] found significant relationship between gender and sign comprehension, but others did not [8,19,37,45,46]. Unfortunately, there was only one female subject in the present study, so the influence of gender on comprehension could not be examined.

Some previous studies [[1], [2], [3], [4], [5], [6]] indicated that older participants performed significantly worse than younger participants. On the other hand, some other studies [8,39,47,48] found no relationship between age and sign comprehension. The ANOVA test in the present study did not find any significant difference among age groups (p = 0.270), although average comprehension performance increased with age (62.73% for 25 years or less, 66.47% for 26–30 years, 66.47% for 31–35 years, 67.04% for 36–40 years, and 76.54% for 41 years or older).

An independent-samples *t*-test revealed that there was a significant difference (p = 0.023) between the average performance of employees with 10 years or less experience and employees with more than 10 years of experience. Subjects with 10 years or less experience (n = 101) performed worse (65.62% average with 14.31% standard deviation) than subjects with more than 10 years of experience (n = 38) (70.97% average with 10.34% standard deviation). Chi-square tests also revealed that correct response to the meaning of the sign was significantly influenced by employee experience, as suggested by Refs. [24,33,49]. Employees with at least 10 years of experience showed better comprehension on the signs "No leaning against" (P041, p = 0.032), "Machinery may start automatically" (W018, p = 0.021), and "Do not extinguish with water" (P011, p = 0.035) than employees with 10 years or less experience.

There was a statistically significant effect of job position (ANOVA, p = 0.023) on comprehension performance. Managers, engineers, technicians, operators, and other employees scored on average 67.29%, 71.43%, 71.86%, 65.05%, and 55.30%, respectively. There are similar results in the literature. Tam et al. [41] found that there were significant differences between worker and site staff.

Finally, educational level was significant (independent samples, p = 0.006) for comprehension performance, as suggested by some previous studies [7,24,47]. Employees (n = 35) with at least a bachelor's degree comprehend the signs significantly better (72.12%, SD = 11.93%) than the group of employees (n = 99) with an elementary school, high school, or associate's degree (65.31%, SD = 13.51%). At the individual sign level, the chi-square test revealed that employees with at least a bachelor's degree correctly answered the meaning of the sign "Break to obtain access" (E008) compared to their counterparts (p = 0.008).

In the legally required safety training for all employees, they learn the meaning of the color and shape of a sign. In the present study, 95% of the subjects reported that they received this safety training. This means that employees should know the type and purpose of the sign. However, the distribution of responses indicates that subjects either do not know the meaning of the color and shape of the signs or that the pictogram depicted on the sign conveys more information than its color and shape. For example, 15.3% of subjects responded to the sign "Break to obtain access" (E008) with "Holding the bar is prohibited." If they knew that the red color and circular shape meant a prohibition, they would not choose the green color and rectangular shape as a prohibition sign. In the same way, "Location of an automated external heart defibrillator device" (E010) is an emergency sign, but 14.8% of subjects think it stands for a prohibition ("No access for people with active implanted cardiac device"). Previous studies [3,[10], [11], [12], [13]] have shown that there is a significant association between training and better comprehension level; therefore, employee training is strongly recommended. In addition, the effectiveness of this training should be evaluated.

## 4 Conclusion

To ensure that safety signs are comprehended by an effective percentage of the population, two standards are typically used (a minimum comprehension score of 67% by ISO 3864 and 85% by ANSI-Z535). In the present study, thirteen safety signs (59% of all signs) met the ISO criterion, but only five signs (23% of all signs) met both the ISO and ANSI criteria to be considered acceptable. The prohibition signs were the best comprehended (86.8% on average) and the hazard signs the worst (53.2%), as suggested in the literature [2].

The comprehension score of four signs was very poor: "Toxic material" (W016, 22.5%), "Location of an automated external heart defibrillator device" (E010, 27.7%), "Overhead obstacle" (W020, 35.0%), and "Disconnect mains plug from the electrical outlet" (M006, 38.8%). The results show that the design of safety signs can confuse employees, leading to occupational injuries and illnesses. It is not essential that the entire workforce recognize all safety signs. However, it is very important that they are comprehended by most employees. It should be noted that there is a possibility that a disaster could be caused by a simple mistake, such as only one person misunderstanding the meaning of a sign).

The present study suggests that some personal factors influence the level of comprehension. Subjects with at least 10 years of experience performed better than subjects with 10 years or less experience. There were also significant differences among job positions. Engineers and technicians had the highest comprehension performances. In addition, education level had a statistically significant effect on compression performance. Subjects with at least a bachelor's degree performed significantly better than subjects with an elementary school, high school, or associate's degree. On the other hand, there was insufficient evidence for an age effect on comprehension performance. This could be due to the fact that the number of oldest participants is small. Alternatively, the lack of an age difference could be due to the greater experience of the older workers.

In the present study, the comprehension level was assessed. However, the reaction time is also important. Seconds can save lives, that is why it is important to get the correct meaning of a sign as quickly as possible. Future studies can investigate the comprehension level as well as the reaction time to make the decision. Besides, in the present study, the occurrence of safety sign in their workplaces was not observed. Subjects were not asked to rate the occurrence of safety sign in their workplaces, either. For example, a poorly designed sign might have a high comprehension score if the employee exposure is high. Future studies may take into consideration the number of signs in their studies to assess the comprehension level.

According to the ISO 7010, safety signs are designed to be universally understood through pictograms. Signs should not contain text because the pictograms on a sign should be comprehensible regardless of the language proficiency of the person viewing the sign. If needed, text may be included on a supplementary sign board, which will provide clarity and additional information. Conversely, the ANSI standard recommends using signal words (danger, warning, caution, and notice) and safety words (written message) in addition to pictograms to convey more specific details to the viewer. Legibility refers to how easily the content of a sign can be read and understood by the intended audience. There are several factors that affect the legibility a sign, such as its size and placement. The size of a sign is a critical element in its legibility, as a sign that is too small can be difficult to read from a distance, while a sign that is too large can be overwhelming. The optimal size of a sign depends on factors such as viewing distance and the amount of information it is intended to convey. This is especially important for warnings of hazards that pose a threat even from a distance, such as radioactivity or biohazard, but less so for drinking water location. Placement of signs is also critical to their legibility, and signs should be placed in a location where they can be easily seen by the intended audience. This may require careful consideration of factors such as lighting, obstacles, and viewing angles. Although the present study focused solely on comprehension, it may be important for future research to take into account legibility as another factor for symbol signs.

The present study suggests that a redesign process for low comprehension signs could be considered. Based on the study results, it can be assumed that subjects pay more attention to the pictogram on the sign than to the color and shape of the sign. It can be suggested that safety sign designers should focus on the pictograms to achieve better comprehension of the safety signs. Comprehension of a sign can be enhanced when it contains some details and depicts real objects [32]. Safety signs should be designed with input from engineers, designers and human factors experts. The opinion of employees should be taken into account in the redesign, as well.

Methods and materials used in safety sign training are also important. A user-centered design approach of safety training materials can increase learning effectiveness [50]. This is particularly correct when there are communication barriers due to language and cultural differences [51]. Proper understanding of the signs can be achieved by designing special and frequent training to better recognize the safety sign of employees according to their ethnic and cultural backgrounds. Designing specific and frequent training also serves a reminder function [32]. Targeted training interventions promote early detection of hazards and associated risks [52].

## Author contribution statement

Celal Gungor, Ph.D.: Conceived and designed the experiments; Performed the experiments; Analyzed and interpreted the data; Contributed reagents, materials, analysis tools or data; Wrote the paper.

## Data availability statement

Data included in article/supp. material/referenced in article.

## Ethics statement

The study was approved by the Social Sciences Ethics Committee of Izmir Katip Celebi University (SAE -0215-2021/23-01). All ethical principles, codes, and guidelines of the Belmont Report, the Nuremberg Code, and the Declaration of Helsinki were carefully followed by the researcher to protect the free will, privacy, confidentiality, and well-being of the subjects.

## Declaration of competing interest

The authors declare that they have no known competing financial interests or personal relationships that could have appeared to influence the work reported in this paper.
