# Supplementary material for: Safety sign comprehension of fiberboard industry employees
Source: Heliyon. 2023 May 26;9(6):e16744. doi: 10.1016/j.heliyon.2023.e16744 (PMC10258412; doi:10.1016/j.heliyon.2023.e16744)
Supplement: Multimedia component 1 [file mmc1.docx]

**Safety Signs Questionnaire Survey**

**Demographics**

We invite you to participate in the research titled "Safety and Health Signs Awareness Level." Before deciding whether or not to participate in this research, it is important that you know why and how the research will be conducted. Therefore, it is of great importance that you read and understand this form thoroughly before participating in the study. If it contains points that you do not understand and are not clear to you, or if you would like more information, please ask.

Participation in this study is completely voluntary. You have the right not to participate in the study or to exit at any time or immediately after participation. Your response to the study will be interpreted as your consent and voluntary participation in the study. While answering the questions on the forms given to you, do not accept pressure from anyone or be under suggestion. ***Your personal information that you will declare in these forms will be kept completely confidential and will be used for research purposes only.***

1. Research Related Information:
   1. Purpose of Research: To determine the effectiveness of health and safety signs
   2. Content of the Survey: A questionnaire consisting of health and safety signs
   3. Reason for Research: Original research
   4. Projected Duration of the Study: About 6 months
   5. Number of Participants/Volunteers Expected to Participate in the Research: 1,000
   6. Researcher: Celal GÜNGÖR celal.gungor@ikcu.edu.tr
   7. Research Place(s): Different sectors
2. **Consent Form to Participate in the Study:**

I have read the information above and should be given to the participant/volunteer before the research and I fully understand the scope and purpose of the study I am asked to participate in, and my voluntarily responsibilities. Written and verbal explanations regarding the study to be carried out were made by the researcher whose name and surname are written below, during which I had the opportunity to ask questions and discuss and received satisfactory answers. The possible risks and benefits of the study were also verbally explained to me. I understood that I could leave this work whenever I wanted and without having to give any reason, and that I would not face any negative consequences if I quit. In these circumstances, I accept to participate in the research in question of my own decision and will, without pressure, coercion or suggestion of any other person.

**Name-Surname:**

**Date:**

**Signature:**

**1.** Your gender:

🞏 Female 🞏 Male

**2.** Your age:

**3.** What is the highest level of education you have completed?

🞏 Elementary school

🞏 High school

🞏 Associate’s degree

🞏 Bachelor’s degree

🞏 Master’s degree

🞏 Doctorate degree

**4.** What is your position?

🞏 Manager

🞏 Engineer (engineer, chief or senior engineer)

🞏 Technician (foreman, mechanic, etc.)

🞏 Operator (maintenance, production, etc.)

🞏 Other employee (general service employees etc.)

**5.** Your total work experience:

**6.** Have you ever received training on health and safety signs?

🞏 Yes, I have received training in the workplace.

🞏 Yes, I had received an additional training in order to sit for the Occupational Health and Safety Expert exam.

🞏 I have received some type of training other than these trainings.

🞏 No, I have not had any training at all.

**7.** How would you describe your level of knowledge about occupational health and safety?

🞏 1: Very little knowledge level

🞏 2: Little knowledge level

🞏 3: Medium knowledge level

🞏 4: High knowledge level

🞏 5: Very high knowledge level

| 1 | 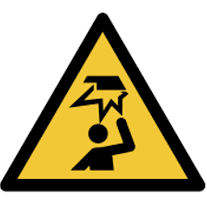 | a. | Do not replace the bulb unless you are authorized |  | 8 | 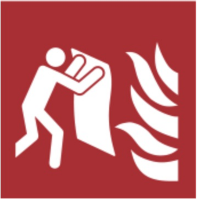 | a. | Prevent the spread of fire |
| --- | --- | --- | --- | --- | --- | --- | --- | --- |
|  |  | b. | Excessive light source |  |  |  | b. | Protect yourself from fire |
|  |  | c. | Falling object hazard |  |  |  | c. | Location of a fire blanket |
|  |  | d. | Overhead obstacle |  |  |  | d. | It is forbidden to approach the fire area |
|  |  |  |  |  |  |  |  |  |
| 2 | 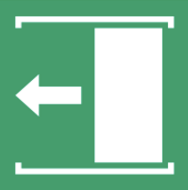 | a. | Slide equipment to move |  | 9 | 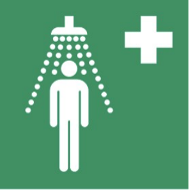 | a. | Take a shower before entering the building |
|  |  | b. | Refrigerator can move |  |  |  | b. | Location of a safety shower |
|  |  | c. | Door slides left to open |  |  |  | c. | Do not use this device in a bathtub, shower, or water-filled reservoir |
|  |  | d. | Exit door is on the left side |  |  |  | d. | Caution, you may get wet |
|  |  |  |  |  |  |  |  |  |
| 3 | 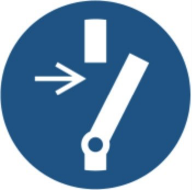 | a. | Disconnecting the machine or equipment before carrying out maintenance or repair |  | 10 | 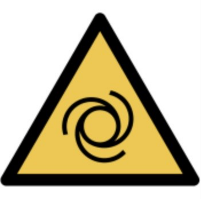 | a. | Low temperature/freezing conditions |
|  |  | b. | The train changes track |  |  |  | b. | Machinery may start automatically |
|  |  | c. | Pull the handle to stop |  |  |  | c. | Heavy wind |
|  |  | d. | Change the line |  |  |  | d. | Biological hazard |
|  |  |  |  |  |  |  |  |  |
| 4 | 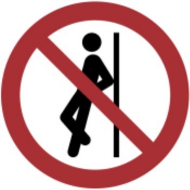 | a. | Go back to work |  | 11 | 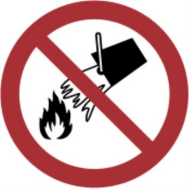 | a. | No open fire |
|  |  | b. | No pushing |  |  |  | b. | No fire |
|  |  | c. | Waiting is forbidden here |  |  |  | c. | Do not extinguish with water |
|  |  | d. | No leaning against |  |  |  | d. | Do not extinguish the fire |
|  |  |  |  |  |  |  |  |  |
| 5 | 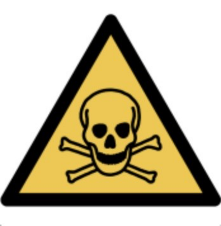 | a. | Electrical hazard |  | 12 | 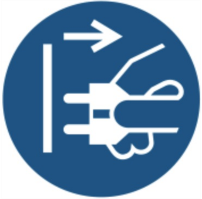 | a. | It is forbidden to insert the plug into the outlet |
|  |  | b. | Danger of death |  |  |  | b. | Disconnect mains plug from the electrical outlet |
|  |  | c. | High voltage |  |  |  | c. | When pulling the plug from the outlet, pull the plug, not the cord |
|  |  | d. | Toxic material |  |  |  | d. | Disconnect the plug before carrying out maintenance or repair |
|  |  |  |  |  |  |  |  |  |
| 6 | 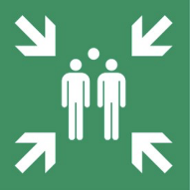 | a. | Evacuation assembly point |  | 13 | 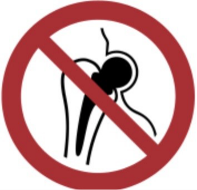 | a. | Choking hazard |
|  |  | b. | Attention, there are people nearby |  |  |  | b. | No reaching in |
|  |  | c. | Entry is prohibited. Only certain people can enter. |  |  |  | c. | No access for people with metallic implants |
|  |  | d. | Enter with a group |  |  |  | d. | Crushing of hands |
|  |  |  |  |  |  |  |  |  |
| 7 | 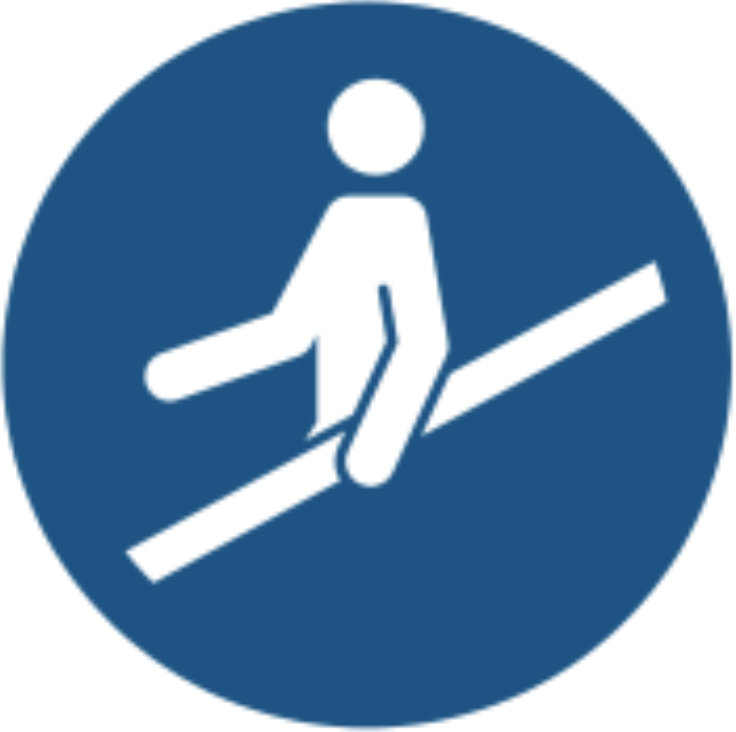 | a. | Stairs |  | 14 | 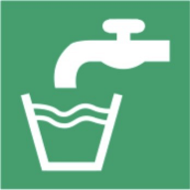 | a. | Turn off the tap after use |
|  |  | b. | Escalator |  |  |  | b. | Hot water |
|  |  | c. | Use handrail |  |  |  | c. | Location of drinking water |
|  |  | d. | Inclined road |  |  |  | d. | Not drinking water |
|  |  |  |  |  |  |  |  |  |
| 15 | 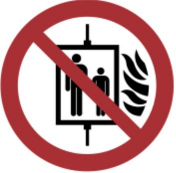 | a. | Do not use lift in the event of fire |  | 20 | 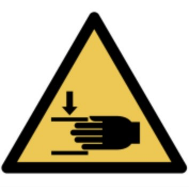 | a. | No reaching in |
|  |  | b. | Do not use the lift for more than one person |  |  |  | b. | Put your hand inside |
|  |  | c. | Do not use the lift for people |  |  |  | c. | Do not touch |
|  |  | d. | Do not approach with open flame |  |  |  | d. | Crushing of hands |
|  |  |  |  |  |  |  |  |  |
| 16 | 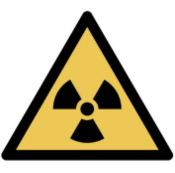 | a. | Aircraft flight area |  | 21 | 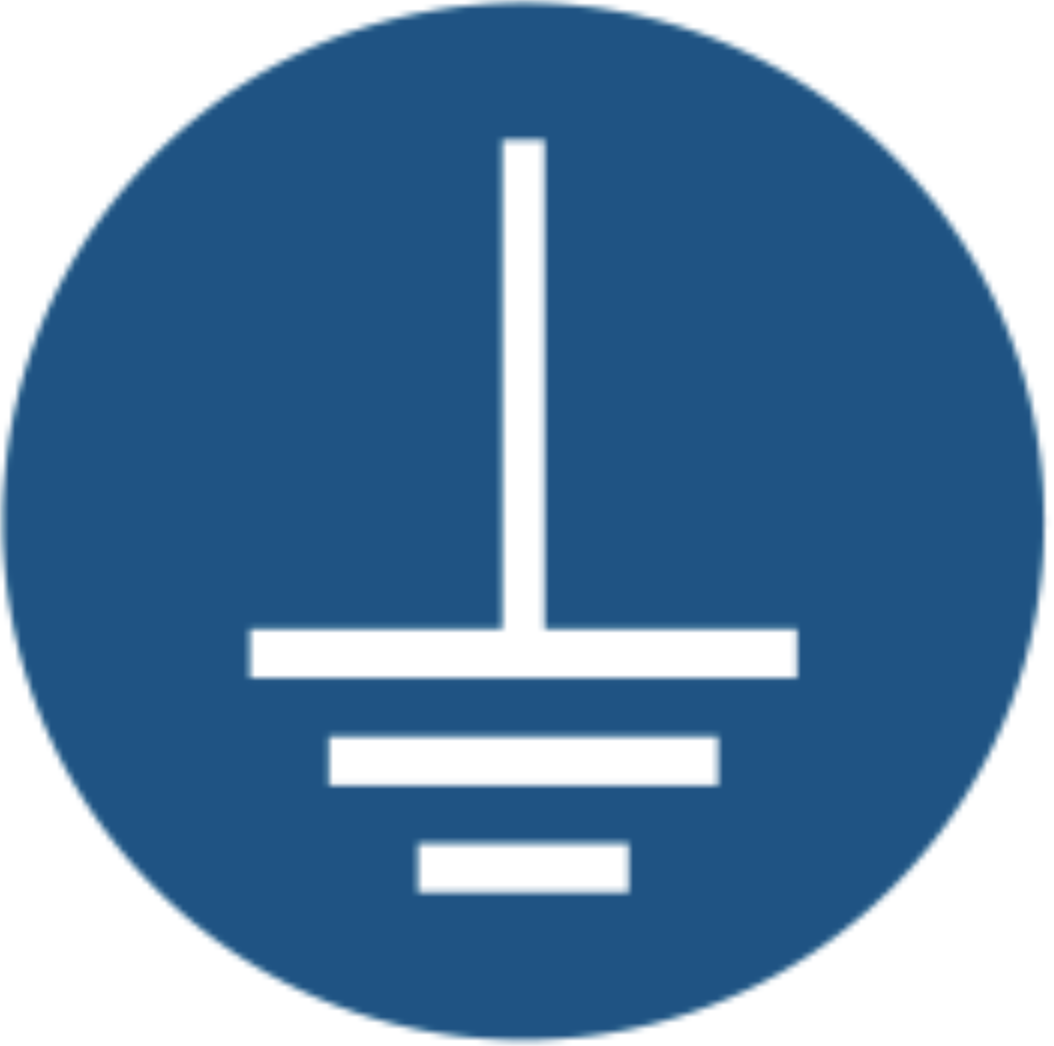 | a. | Connect an earth terminal to the ground |
|  |  | b. | Chemical spill hazard |  |  |  | b. | Heavy load (dumbbells) |
|  |  | c. | Radioactive material or ionizing radiation |  |  |  | c. | High radiation area |
|  |  | d. | Biological hazard |  |  |  | d. | Strong signal area |
|  |  |  |  |  |  |  |  |  |
| 17 | 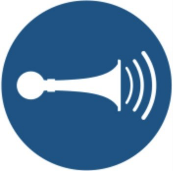 | a. | Sound horn |  | 22 | 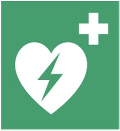 | a. | Location of an automated external heart defibrillator device |
|  |  | b. | Do not sound horn |  |  |  | b. | High voltage power can cause your heart to stop |
|  |  | c. | Caution, very high horn sound |  |  |  | c. | No access for people with active implanted cardiac devices |
|  |  | d. | Horn can be sounded at certain times |  |  |  | d. | Check the power level of your implanted cardiac device |
|  |  |  |  |  |  |  |  |  |
| 18 | 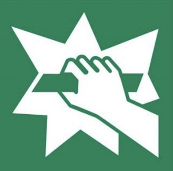 | a. | Clear (clean) the glass |  |  |  |  |  |
|  |  | b. | Glass breakage hazard |  |  |  |  |  |
|  |  | c. | Holding the bar is prohibited |  |  |  |  |  |
|  |  | d. | Break to obtain access |  |  |  |  |  |
|  |  |  |  |  |  |  |  |  |
| 19 | 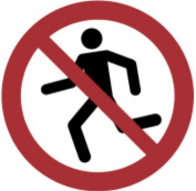 | a. | Do not run |  |  |  |  |  |
|  |  | b. | No pedestrian |  |  |  |  |  |
|  |  | c. | No thoroughfare/trespassing |  |  |  |  |  |
|  |  | d. | Use this walkway |  |  |  |  |  |
